# What Distinguishes Women Who Choose to Self-Inject? A Prospective Cohort Study of Subcutaneous Depot Medroxyprogesterone Acetate Users in Ghana

**DOI:** 10.9745/GHSP-D-21-00534

**Published:** 2022-02-28

**Authors:** Dela Nai, Elizabeth Tobey, Kamil Fuseini, Patrick Kuma-Aboagye, Aparna Jain

**Affiliations:** a Population Council, Accra, Ghana.; b Population Council, Washington, DC, USA.; c Ghana Health Service, Accra, Ghana.

## Abstract

Depot medroxyprogesterone acetate administered subcutaneously (DMPA-SC) is an all-in-one injectable contraceptive administered every 3 months, either by a trained health care provider or community health worker or by training a client to self-inject. Using a prospective cohort of family planning (FP) clients in Ghana, this study explores patterns of DMPA-SC use and mode of injection administration over a 6-month period. This study also examines the predictors of self-injection adoption 6 months after initiating DMPA-SC. Our analysis focuses on 378 women who were using DMPA-SC at the 6-month interview. Adjusted odds ratios accounting for clustering show that clients who were new FP users, never married, or attended high school/attained higher education were significantly more likely to self-inject by the third injection. Results of this study suggest that in Ghana, adding DMPA-SC to the method mix may improve access to FP, especially among new users. Results of this study may inform FP projects and programs aiming to improve access to contraceptive methods and increase contraceptive prevalence by introducing or scaling up DMPA-SC self-injection. The findings also provide a sociodemographic profile of FP clients most likely to adopt DMPA-SC self-injection over time, which could serve as an evidence base for social marketing strategies.

## Abstract (precis)

Family planning projects and programs seeking to introduce, scale up, or market subcutaneous depot medroxyprogesterone acetate self-injection should first focus efforts on new family planning users, those never married, and those with at least a high school education level.

## Teaser

**Key Findings:**

- At their third injection, or 6 months into using subcutaneous depot medroxyprogesterone acetate (DMPA-SC), 73% of these users chose self-injection, an increase from 42% who chose self-injection at the first injection.
- Clients who were new family planning users, never married, or attended high school/attained higher education were significantly more likely to self-inject by the third injection compared to their respective counterparts. Expanding method choice through this new mode of injection administration may help to meet these users’ needs.

**Key Implications:**

- Policy makers and health practitioners in Ghana and other countries with similar profiles should consider results of this study on the key characteristics of DMPA-SC users, patterns of use and mode of injection administration, and factors that increase the likelihood of self-injection when making evidence-based decisions regarding DMPA-SC introduction and/or scale-up.
- When given the option to choose between provider-administered injection and self-injection at each injection opportunity, many women will learn to self-inject and the path to self-injection may be gradual. Policy makers and health practitioners should consider presenting both choices of administration of injection at every opportunity.

## INTRODUCTION

In Ghana, between 22% and 25% of married women use a modern contraceptive method, and close to a third (32%) of sexually active unmarried women do so.^1^^,^^2^ The total fertility rate (TFR) ranges between 3.9 and 4.2 births per woman.^1^^,^^2^ These data also reveal that approximately 30% of married women in Ghana wanting either no more children or to postpone childbearing for at least the next 2 years were not using any contraceptive method.^1^ To meet the needs of these women who want to delay or avoid childbearing, exploring new avenues for increasing access to modern family planning (FP) methods is paramount.

The proportion of currently married contraceptive users in Ghana using an injectable contraceptive has increased from 2% in 1988 to 27% in 2017,^2^ and it is the most popular modern contraceptive method used among this group. The most common type of contraceptive injectable contains depot medroxyprogesterone acetate (DMPA) as the active ingredient and is administered intramuscularly (IM), using a syringe and a vial. The subcutaneous (SC) version of the DMPA injectable contraceptive is an all-in-one product that combines a single dose of the drug and a needle using the Uniject injection system. DMPA-SC can be administered either by a trained health care provider/community health worker or by training the FP client to self-inject. DMPA-SC and DMPA-IM are similar in that they are both safe, 99% effective, administered every 3 months, and have similar side effect profiles.^3^ However, DMPA-SC has a shorter needle (1 cm) and a lower dosage (104 mg) compared to DMPA-IM (2.5 cm and 150 mg, respectively).

In Africa, 4 countries were among the first to introduce DMPA-SC (between July 2014 and June 2016) and to document their experiences: Burkina Faso,^4^^,^^5^ Niger, Senegal, and Uganda,^5^ all showing promising results. Nevertheless, before 2017, there was limited empirical evidence on the feasibility and acceptability of DMPA-SC self-injection among women in Africa. A qualitative study on the perceptions of home and self-injection of DMPA-SC in Ethiopia showed that after watching a demonstration and testing the Uniject on a nonhuman model, study participants enjoyed the simple design of the product, found it easy to use, and appreciated the time and savings that home self-injection would offer.^6^ In Senegal, a prospective cohort study found that 87% of the study population self-injected competently 3 months after being trained by a provider, and 93% of those who self-injected expressed the desire to continue self-injecting.^7^ A similar study conducted in Uganda also found high feasibility and acceptability of DMPA-SC self-injection.^8^ A 12-month randomized control trial to measure the effects of self-injection versus provider-administered injection among women in Malawi demonstrated that self-injection improved DMPA-SC continuation rates when compared with provider-administered injections.^9^

In 2016, the Ghana Health Service (GHS) convened a technical advisory group to undertake an in-country implementation science study to broaden the evidence base on the feasibility and acceptability of DMPA-SC self-injection and in a context where DMPA-SC and self-injection were being introduced simultaneously. Beginning in late December 2017, a feasibility and acceptability study was conducted in Ashanti and Volta regions and a prospective cohort of DMPA-SC initiators was followed over 6 months (the equivalent of 9 months of contraceptive coverage), allowing for examination of 3 injections. Our analyses use data from the pilot study to explore patterns of DMPA-SC use and mode of injection administration throughout the period and to examine the predictors of DMPA-SC self-injection at the third injection. Understanding the profile of DMPA-SC users, patterns of use, and predictors of self-injection can help inform FP programs that seek to increase choice and access to FP methods, reduce unmet need, and increase the modern contraceptive prevalence rate (mCPR).

Understanding the profile of DMPA-SC users, patterns of use, and predictors of self-injection can help inform FP programs that seek to increase choice and access to FP methods.

## PROGRAM DESCRIPTION

### Study Setting

The study was implemented in public health facilities in Ashanti and Volta regions. The Ashanti region is the most populous region of Ghana (approximately 5 million residents), with a TFR of 4.4 births per woman, an mCPR of 21%, and approximately 6% of married women using an injectable.^1^ The Volta region has a population of 3 million residents, a TFR of 3.6 births per woman, an mCPR of 30%, and 14% of married women using an injectable.^1^ The Ashanti and Volta regions are noncontiguous and are both predominantly rural.

### Intervention Description

The technical advisory group, represented by private-sector, public-sector, and donor institutions, selected the study’s intervention regions and districts. In each region, 4 public health facilities were selected across 4 districts based on their location: 2 rural, 1 periurban, and 1 urban, as defined by GHS. In addition to their location, facilities were eligible for the study based on their monthly caseload of injectable users and tracking of FP service statistics using rsLog (a health management information system piloted by GHS).

A master trainer from PATH Uganda led a 4-day training for 8 GHS regional resource trainers. The training provided an overview of DMPA-IM and DMPA-SC, features of the Uniject device, counseling information including side effects management and follow-up options for each method, and steps to administer each method. Regional resource trainers were trained on how to train providers to train clients on the following aspects of DMPA-SC: (1) self-injection technique; (2) safe storage; (3) safe disposal of used devices in a puncture-proof container; and (4) calculating reinjection dates. The training also included role playing and nonhuman models to practice provider-administered injection and client self-injection. Regional resource trainers went on to train 150 FP health care providers across study facilities (71 in Ashanti Region and 79 in Volta Region), including community health nurses, enrolled nurses, and midwives. The training covered the aforementioned topics and used the same practice/learning methods. Each trained provider was given a set of DMPA-SC job aids (developed by PATH). The trained providers, in turn, counseled FP clients at the facility on all available contraceptive methods, including DMPA-SC and its 2 modes of injection administration. For clients who chose DMPA-SC, providers assessed their eligibility to receive the method using the World Health Organization’s Medical Eligibility Criteria for contraceptive use.^10^

Providers offered clients who chose DMPA-SC the option to either receive the injection by the provider or be trained by the provider to self-inject. If a client chose to self-inject, she was trained and allowed to practice self-injection up to 5 times on a condom filled with salt, which mimicked fat under the skin. Using an observational checklist and their clinical judgment, the provider assessed the clients’ ability to self-inject safely after the client successfully executed 5 of 11 critical self-injection steps (Box, with critical steps bolded). Following successful self-injection practice, the client then self-injected DMPA-SC in front of the provider. Clients who self-injected at the facility (“on-site self-injection”) were given up to 2 DMPA-SC doses for home self-injection, along with a self-injection instruction sheet, a puncture-proof container, and a 2017–2018 reinjection calendar with dates for the next 2 injections circled. Clients who chose provider-administered injection and did not practice self-injection or those who did not adequately demonstrate the ability to self-inject were told about their follow-up visit. The date for the next visit was indicated on their FP card per usual practice. A full description of the intervention can be found elsewhere.^11^

BOX **Checklist^a^ for DMPA-SC Self-Injection Practice**

Step 1: Washes hands.

**Step 2: Selects an appropriate injection site and cleans it if needed.**

Step 3: Opens the Sayana Press pouch by tearing from the notch.

**Step 4: Mixes the liquid by shaking the device vigorously for about 30 seconds.**

**Step 5: Pushes the needle cap and port together to activate the device.**

Step 6: Removes the needle cap.

**Step 7: Pinches the “skin” at the injection site to form a “tent.”**

Step 8: Inserts the needle completely so that the port is in full contact with the skin.

**Step 9: Presses the reservoir slowly to inject for about 5 to 7 seconds.**

Step 10: Removes the device from the injection site while still pinching the skin.

Step 11: Immediately places the used device in a sharps disposal container without replacing the needle cap.

^a^ Bold indicates critical steps.

## METHODS

The prospective cohort study was conducted with newly initiating DMPA-SC clients followed over a 6-month period, allowing for 3 injection times and 3 rounds of interviews. Clients eligible for the prospective cohort study included women aged 18–49 years who sought FP services from 1 of the 8 study facilities, were not planning on becoming pregnant in the next 6 months, and were more than 6 weeks postpartum or breastfeeding. Eligible clients were also willing to provide a phone number and to be reached by phone for follow-up interviews. After receiving their injection (provider-administered or self-injected), providers informed clients who chose DMPA-SC about the pilot study, and those who chose to learn more about the study were introduced to a research team member on-site for the informed consent and first interview. The first round of interviews was all in-person and took place between December 2017 and January 2018. We primarily conducted telephone interviews 3 months (March 2018–April 2018) and 6 months (June 2018–July 2018) later, which corresponded to the client’s scheduled second and third injections, respectively. In some cases, these subsequent interviews were conducted in person where phone connectivity was an issue or if the client expressed a strong preference for in-person interviews.

A total of 568 women completed their first interview after their first injection. The analysis in this article focuses on 378 women who were using DMPA-SC at the third interview (after the third injection) and excludes 68 women who discontinued using DMPA-SC and 122 who were lost to follow-up. We collected information on sociodemographic characteristics, previous use of FP, awareness of DMPA-SC, quality of care received, experiences with DMPA-SC counseling, training, and administration, and reporting of serious adverse reactions. Additional questions in the second and third interviews included experiences with most recent injection, experience of side effects, and for those who self-injected at home, experiences with home self-injection, reinjection dates, storage, and disposal of DMPA-SC. Clients who were reached for the second and third interviews but had discontinued DMPA-SC or withdrawn from the study were asked to complete a short survey about their reasons for discontinuing or withdrawing.

### Ethics Approval

The study received ethical approval from the Population Council Institutional Review Board and the GHS Ethical Review Committee. All participants gave their written informed consent before the first interview. Before the second and third interviews, respectively, all participants gave their verbal informed consent. Interviews were conducted in English or the local language (per the participant’s preference) in a space chosen by the participant and one that ensured privacy and confidentiality of personal information.

### Variables

The dependent variable for this analysis is self-injection of DMPA-SC for the third injection, where self-injection could take place either at home or at the facility. At the first interview, women were asked how their injection was administered, to which response options were “provider-administered” or “self-administered.” Those who reported self-administration were classified as self-injectors and coded as 1, while women who chose provider administration were coded as 0. Additionally, at the first interview, women who reported that they had self-injected and that they were given at least 2 DMPA-SC units to take home were classified as potential home self-injectors. At the second and third interviews, clients were asked the same set of questions to determine their mode of injection category.

Of interest were several independent variables collected at the first interview: age, marital status (never married or ever married/in union, which comprised those currently married, living together, separated, divorced, or widowed), number of living children, education (no education, primary, junior secondary/high school, senior secondary/high school and above), employment status (currently working or not currently working), previous use of FP (first-time user, previous DMPA-IM user, previous other FP method user) and receipt of MIIplus (complete MIIplus or incomplete MIIplus). The Method Information Index (MII) is an FP indicator of informed choice created by FP2020.^12^ It is routinely collected in the Demographic and Health Surveys (DHS) and consists of 3 questions related to women's reports about the information received at the time of method adoption and the reported value is the percentage of women who responded “yes” to all 3 questions.^12^ Chang et al. explain that the aim of verifying that the client received complete information about her FP method is not only to ensure informed choice but also to ensure contraceptive continuation.^13^ To this end, Jain et al. conducted a study in which they added a fourth question to the MII related to the possibility of switching to a different contraceptive method in the event that the current one was not suitable and found that the addition of this fourth question better predicts contraceptive continuation than the MII alone. The revised MII is referred to as MIIplus.^14^

To account for similarities between women who received their method at the same or other study facilities, a variable combining region and residence was created, with the categories urban Volta, rural Volta, urban Ashanti, and rural Ashanti. The rural/urban location was that of the facility from which the woman obtained her method, as defined by GHS. Each category of the region/residence variable comprised 2 facilities. Additional information is available elsewhere.^11^ This variable was used to adjust the multivariate model for random effects at the regional/residence level since earlier analyses (not shown) using the health facility level perfectly predicted self-injection for one of the facilities.

### Analysis

Descriptive analyses were conducted for the dependent and independent variables of interest. Additionally, bivariate analyses were conducted to examine self-injection of DMPA-SC at third injection by independent variables, using Pearson’s chi-squared tests for significance. A multivariate logistic regression model with random effects to account for clustering at the region/residence level was used to assess the relationship between independent variables and self-injection of DMPA-SC at the third injection. Independent variables were included in the model based on theoretical importance. All analyses were conducted using Stata version 15 (Statacorp). Sankey diagrams were created to visualize patterns in DMPA-SC use, mode of administration, and study participation over the course of the study, both for the analytic sample of interest and the original study sample.

## RESULTS

Table 1 presents the characteristics at baseline of respondents in the analytic sample. One-third of respondents were between 18 and 24 years (33%). More than two-thirds of respondents were ever married or in union (70%) and most had at least 1 child (85%). The majority of respondents lived in urban areas (62%), were currently employed (61%), and exactly half attended junior secondary school/junior high school. Less than half of respondents had never used a contraceptive method in the past (44%) and nearly three-quarters (74%) received the full MIIplus at their visit on the day of their first interview. At their third injection, 73% self-injected DMPA-SC.

**TABLE 1:** Characteristics of Women at First Injection Who Continued Using DMPA-SC at Third Injection, Ghana (n=378)

	No. (%)
Age range, years	
18–24	124 (32.8)
25–29	98 (25.9)
30–34	72 (19.0)
35 and older	84 (22.2)
Marital status	
Never married	114 (30.2)
Ever married/in union	264 (69.8)
Number of living children	
None	57 (15.1)
1-2	162 (42.9)
3+	159 (42.1)
Region/residence	
Ashanti rural	67 (17.7)
Ashanti urban	93 (24.6)
Volta rural	77 (20.4)
Volta urban	141 (37.3)
Education	
No education	31 (8.2)
Primary	70 (18.5)
Junior secondary school/junior high school	189 (50.0)
Senior secondary school/senior high school or higher	88 (23.3)
Employment status	
Not currently working	148 (39.2)
Currently working	230 (60.8)
Previously used FP	
New FP user	166 (43.9)
Previous DMPA-IM user	172 (45.5)
Previous user of other method	40 (10.6)
Receipt of MIIplus	
Did not receive full MIIplus	98 (25.9)
Received full MIIplus	280 (74.1)
Mode of administration of third injection	
Provider-administered	104 (27.5)
Self-administered	274 (72.5)
At the facility (on-site)	96 (25.4)
At home	178 (47.1)

Abbreviations: DMPA-IM, intramuscular injection of depot medroxyprogesterone acetate; DMPA-SC, subcutaneous injection of depot medroxyprogesterone acetate; FP, family planning; MIIplus, revised method information index.

The Figure presents a Sankey diagram demonstrating patterns of DMPA-SC use, mode of administration, and study participation among the analytic sample of 378 women. At the first injection, 58% of respondents chose provider administration and 42% chose on-site self-injection. At the second injection, a higher proportion of respondents chose self-injection either on-site (22%) or at home (43%), and these proportions increased to a total of 72.5% at the third injection. Of the 159 self-injectors at first injection, 8 women (5%) reverted from on-site self-injection to provider administration at second injection (data not shown), although none who chose home self-injection at the second injection reverted to either provider-administered or on-site self-injection at the third injection. The Figure reveals that among this analytic sample, the most likely outcome for on-site self-injectors was home self-injection and the most likely outcome for those who choose provider administration was on-site self-injection. Approximately one-quarter of the analytic sample remained in the provider administration category throughout the 3 injection times. By the second injection, around 1% of respondents had discontinued DMPA-SC and 8% were lost to follow-up. A Sankey diagram representing patterns of DMPA-SC use, including discontinuation and loss to follow-up among the full study sample is featured in a Supplement.

**FIGURE:**
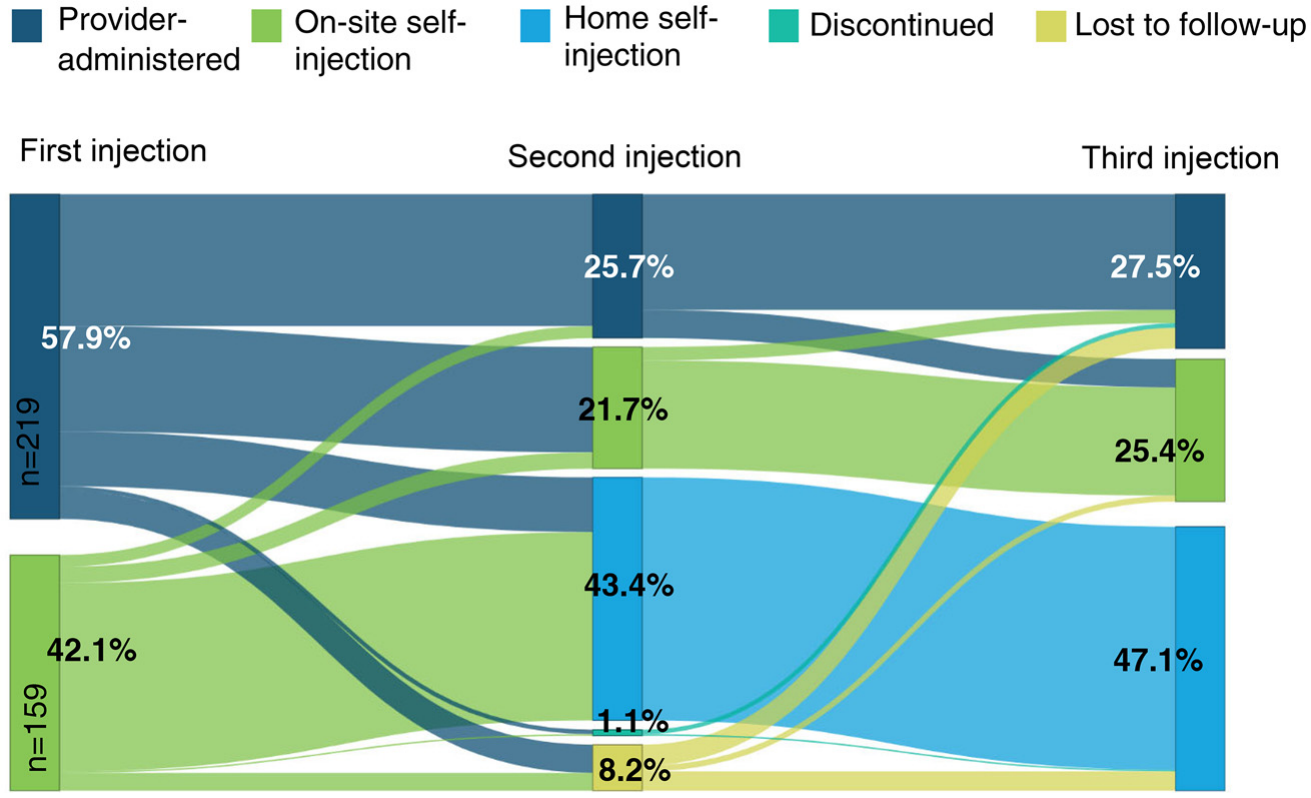
Sankey Diagram of Patterns of DMPA-SC Mode of Injection Administration, Discontinuation, and Lost to Follow-Up Over the 3 Injection Periods (n=378) Abbreviation: DMPA-SC, subcutaneous injection of depot medroxyprogesterone acetate.

Between the first injection and second injection, 37 women moved directly from provider administration to home self-injection, despite study guidelines that clients were to self-inject in the presence of the provider before taking devices home for self-injection. Across the board, providers explained that these clients had successfully practiced self-injection on the model but lost confidence at the last minute, asking the provider to administer the injection. Providers reported giving such clients the 2 doses for home self-injection because they had successfully and sufficiently practiced, and they and the clients deemed that the loss of confidence was temporary.

Table 2 presents the bivariate analyses of mode of injection administration at the third injection, per respondent characteristics. There were significant differences in mode of administration by previous use of FP, as new FP users were most likely to self-inject (81%) whereas those who had previously used DMPA-IM were least likely to self-inject (63%). There were also significant differences by age, as 78% of those aged 18–24 self-injected DMPA-SC, while 58% of those aged 35 and above did so. The likelihood of self-injection was higher among never-married women (81%) compared to those who were ever married/in-union (69%). Those who lived in urban and rural areas of Volta region (85% and 91%, respectively) and in rural areas of Ashanti (82%) were significantly more likely to self-inject than those who lived in urban areas of Ashanti region (31%). Women who received all information of the MIIplus were more likely to self-inject (78%) than those who did not (56%). There were no significant differences in mode of injection administration by number of living children, educational attainment, or employment status.

**TABLE 2.** Mode of DMPA-SC Injection Administration at Third Injection by Respondent Characteristics, Ghana (n=378)

	No.	Provider-Administered, %	Self-Injection, %
Age range, years^a^			
18–24	124	21.8	78.2
25–29	98	25.5	74.5
30–34	72	23.6	76.4
35 and older	84	41.7	58.3
Marital status^a^			
Never married	114	19.3	80.7
Ever married/in union	264	31.1	68.9
Number of living children			
None	57	17.5	82.5
1–2	162	25.9	74.1
3+	159	32.7	67.3
Region/residence			
Ashanti rural	67	17.9	82.1
Ashanti urban	92	68.8	31.2
Volta rural	77	9.1	90.9
Volta urban	141	14.9	85.1
Education			
No education	31	35.5	64.5
Primary	70	21.4	78.6
Junior secondary school/junior high school	189	30.7	69.3
Senior secondary school/senior high school or higher	88	22.7	77.3
Employment status			
Not currently working	148	22.3	77.7
Currently working	230	30.9	69.1
Previously used FP^b^			
New FP user	166	18.7	81.3
Previous DMPA-IM user	172	37.2	62.8
Previous user of other method	40	22.5	77.5
Receipt of MIIplus^c^			
Did not receive full MIIplus	98	43.9	56.1
Received full MIIplus	280	21.8	78.2
Total	378	27.5	72.5

Abbreviations: DMPA-IM, intramuscular injection of depot medroxyprogesterone acetate; DMPA-SC, subcutaneous injection of depot medroxyprogesterone acetate; FP, family planning; MIIplus, revised method information index.

a *P*<.05.

b *P*<.01.

c *P*<.001.

Results of the multivariate logistic regression model of likelihood of self-injection at the third injection, adjusted for clustering at the regional/residence level, are presented in Table 3. Several factors remained significant in the adjusted model. Never-married respondents were 2.32 times more likely to self-inject at the third injection compared to ever-married/in-union women, after adjusting for other respondent characteristics (95% CI=1.05, 5.14). Those who had attended senior secondary school/senior high school or higher were significantly more likely to self-inject than those who had attended junior secondary school/junior high school (AOR: 2.69, 95% CI=1.21, 6.01). Post estimation linear combination tests showed no other differences between educational attainment categories. Furthermore, new FP users were 2.51 times more likely to self-inject DMPA-SC at the third injection than women who were previous users of DMPA-IM (95% CI: 1.31, 4.80). Although those who were previous users of other FP methods had greater odds of self-injection than previous DMPA-IM users, this difference was not statistically significant (AOR: 2.68; 95% CI=0.98, 7.34). Post estimation linear combination tests showed there was no difference between those who were new FP users compared to previous users of methods other than DMPA-IM. Despite its significance in the bivariate analyses, after adjusting by respondent characteristics, there was no significant difference in the odds of self-injection at third injection by receipt of complete information on the MIIplus (AOR 0.85; 95% CI=0.42,1.71). Finally, there were no significant differences by age, number of living children, or employment status.

**TABLE 3.** Adjusted Odds Ratios of DMPA-SC Self-Injection at Third Injection, Ghana (n=378)^a^

	Adjusted Odds Ratio	95% Confidence Interval
Age range, years		
18–24	ref	
25–29	1.23	0.56, 2.69
30–34	1.35	0.49, 3.70
35 and older	0.64	0.25, 1.60
Marital status		
Ever married/in union	ref	
Never married	2.32^b^	1.05, 5.14
Number of living children		
None	Ref	
1–2 children	1.21	0.44, 3.29
3+ children	1.39	0.44, 4.45
Education		
No education	0.93	0.35, 2.50
Primary	1.19	0.55, 2.60
Junior secondary school/junior high school	Ref	
Senior secondary school/senior high school or higher	2.69^b^	1.21, 6.01
Employment status		
Not currently working	Ref	
Currently working	1.58	0.83, 3.00
Previously used family planning		
New FP user	2.51^c^	1.31, 4.80
Previous DMPA-IM user	Ref	
Previous user of other method	2.68	0.98, 7.34
Receipt of MIIplus		
Did not receive full MIIplus	Ref	
Received full MIIplus	0.85	0.42, 1.71

Abbreviations: DMPA-IM, intramuscular injection of depot medroxyprogesterone acetate; DMPA-SC, subcutaneous injection of depot medroxyprogesterone acetate; FP, family planning; MIIplus, revised method information index.

a Adjusted for clustering at the region/residence level.

b *P*<.05.

c *P*<.01.

New FP users were 2.51 times more likely to self-inject DMPA-SC at the third injection than women who were previous users of DMPA-IM.

## DISCUSSION

For countries setting out to introduce and/or scale up DMPA-SC self-injection, understanding key characteristics of DMPA-SC users, patterns of use and mode of injection administration, as well as factors that influence self-injection are all important to make evidence-based decisions. In 2017, the GHS introduced DMPA-SC and self-injection simultaneously and used findings from the pilot study to inform a phased national rollout. While descriptive data have been useful to inform the rollout, more elaborate analyses regarding self-injection could provide refined findings to supplement, enhance, or reorient rollout efforts in Ghana and that of other countries with similar profiles. The present study aims to contribute to our understanding of DMPA-SC users’ characteristics, patterns of DMPA-SC use, and modes of administration, as well as factors that may influence their adoption of self-injection by the third injection.

The recently updated World Health Organization self-care guidelines state that “[T]he use and uptake of self-care interventions is organic and the shift in responsibility—between full responsibility of the user and full responsibility of the health worker (or somewhere along that continuum)—can also change over time for each intervention and for different population groups.”^15^ In its implementation strategy, the GHS was deliberate about offering women the opportunity to choose self-injection over a period of time. This mimicked a routine setting where women can choose their preferred mode of injection administration at a given injection opportunity, as opposed to a study where women are randomized to a provider-administered or self-administered injection. The Sankey diagram we present in the current study provides illustrative evidence of an organic shift and that when given the opportunity, many women will learn to self-inject and the path to self-injection may be gradual. Moreover, the Sankey diagram vividly exemplifies voluntarism and informed choice: at each injection, women were presented with the choice of mode of administration. Voluntary choice of method and informed choice are among the 6 main areas of quality of care,^16^^,^^17^ itself a determinant of uptake and continuation of FP.^18^^,^^19^ The proportion of women in the analytic sample who chose self-injection increased over time, with the majority transitioning to home self-injection. Close to half of those who initiated DMPA-SC with a provider-administered injection chose to transition to on-site self-injection at the second injection, while one-quarter opted to maintain provider administration throughout the study period. Although none of the women who chose self-injection opted for provider-administered injection for subsequent injections, the option to do so remained. Opportunities to re-engage with the health system must be built into the continuity of care for DMPA-SC self-injectors, by emphasizing open access to service delivery points for resupply and disposal, management of side effects, and switching contraceptive methods, among others. The results of our study strongly support the notion that self-care interventions are likely to provide more opportunities for health(care) seekers to make informed decisions, increase choice and autonomy^15^ as well as provide opportunities for health systems to improve equitable access, quality of care, and financial protection for health(care) seekers.^20^

The Sankey diagram provides illustrative evidence of an organic shift and that when given the opportunity, many women will learn to self-inject and the path to self-injection may be gradual.

Our analysis of a prospective cohort of 378 new DMPA-SC users demonstrates that first-time FP users were significantly more likely to self-inject DMPA-SC at the third injection than their counterparts who had used FP before. A reason for this tendency may be that new FP users are attracted to the touted benefits of self-injection, including reduced visits to the facility and increased privacy.^11^^,^^21^^,^^22^ Previous analyses of the DMPA-SC pilot study in Ghana found high acceptability among those who chose home self-injection for the third injection based on satisfaction (98%), comfort (100%), and intention to continue home self-injection (97%). Reasons for opting for home self-injection include not having to travel to the facility (71%), injecting in the comfort of one’s home (53%), and privacy (42%).^11^ The pilot study also found that self-injection clients safely stored and disposed of the Uniject following effective counseling by health providers.^23^ On the other hand, previous FP users may already be used to the routine of their contraceptive method being administered at the facility. The finding suggests that adding DMPA-SC to the method mix in Ghana and offering self-injection as a mode of administration may improve access to FP, especially among new FP users. Expanding method choice by including DMPA-SC and self-injection option to the mix may help to improve contraceptive prevalence, increase the use of more effective contraceptive methods and reduce unwanted pregnancies, all of which respond to Ghana’s FP-related commitments.^24^

The present study also showed that women who had never been married were more likely to self-inject at their third injection, a finding consistent with another study.^25^ Although in the bivariate analyses there were significant differences by age group when comparing those who self-injected and those who did not, age was not associated with self-injection at third injection in the adjusted model. After adjusting for clustering at the regional/residence level, education emerged as an important predictor, with women having completed senior high school or higher being more likely to self-inject at third injection. Although the majority of studies on DMPA-SC conducted in Africa,^9^^,^^25^ including this study in Ghana, have concluded that clients with varying educational and literacy levels can be trained to successfully self-inject, the findings from the present analyses on education are informative from a programmatic perspective, particularly for practitioners who consider market segmentation in their implementation of FP social marketing campaigns as well as decision makers who aim to scale-up DMPA-SC. Of the other characteristics explored in the present study, receiving the complete MIIplus (an indicator of the quality of care received) was associated with self-injection at third injection but this association disappeared in the multivariate analysis. A reason for this could be that MIIplus is assessing the quality of care received as it relates to DMPA-SC and not related to counseling on self-injection, which could be more influential on women’s transition to self-injection.

Our findings from the analyses on education are informative from a programmatic perspective, particularly for practitioners who consider market segmentation in their implementation of FP social marketing campaigns as well as decision makers who aim to scale-up DMPA-SC.

### Limitations

This study is not without limitations. At the third injection, 21.5% (n=122) of respondents were lost to follow-up. The Supplement Table presents findings from a multivariate logistic regression of lost to follow-up at third injection by respondents’ characteristics, excluding respondents who discontinued DMPA-SC because their outcome is known. The majority of characteristics were not significantly associated with being lost to follow-up, with the exception of marital status. This variable was also associated with self-injection. This suggests that the significant association of never-married women being more likely to self-inject at third injection may not be a true effect or may be overestimated.

In addition to population size, the technical advisory group selected Ashanti region based on prior success with marketing of contraceptive methods and Volta region because it had the highest mCPR in the country at the time of the study. Despite the 2 being regions predominantly rural, as are other regions in the country, findings from the present study may not be generalizable to the rest of Ghana. Also, the present study did not consider any client household characteristics that could affect the option to self-inject at home, including having a safe space to store and dispose of the Uniject devices as well as having privacy at home. As such, some clients may find the original option of attending the health facility and getting a provider-administered injection the most suitable. Lastly, the timeliness of the data should also be taken into consideration as data collection ended in 2018 and patterns of DMPA-SC use may have changed over time. These limitations notwithstanding, the present analysis provides useful insights into the characteristics of DMPA-SC self-injectors, their choice to self-inject over time, and factors that play an important choice in self-injection 6 months into using DMPA-SC.

## CONCLUSION

Contraceptive self-injection has been touted to have several advantages, including increased access to FP as well as autonomy and privacy. In a more likely and typical scenario of a non-randomized setting, where FP clients who choose DMPA-SC after counseling are given the choice between a provider-administered injection or self-injection, it is important to examine the profile of women who choose self-injection, their patterns of use, and predictors of this mode of administration. Such information is useful for practitioners who seek to make evidence-based decisions regarding implementation, including social marketing campaigns and national rollouts of DMPA-SC and self-injection. Specifically, in Ghana, where the national rollout of both DMPA-SC and self-injection has begun and has engaged both the public and private sector (with the exception of pharmacies and drug shops), visualizing how DMPA-SC was used over the 6-month period and how women exercised their voluntary choice to self-inject during that period represents a powerful tool and justification for offering women the option of self-injection at any given time. Additionally, understanding that first-time FP users, non-married women, and those with SHS education or higher are all significantly more likely to adopt DMPA-SC self-injection can help to refine segmentation targets or indicators for the remaining portions of the rollout. Other countries that share a similar profile to Ghana can also benefit from the present findings as they embark on their own introductions or scale up of DMPA-SC and/or self-injection.

## Supplementary Material

GHSP-D-21-00534-Supplement.pdf

## Notes

**Peer Reviewed**

**First published online:** February 24, 2022.
