# Supplementary material for: What Distinguishes Women Who Choose to Self-Inject? A Prospective Cohort Study of Subcutaneous Depot Medroxyprogesterone Acetate Users in Ghana
Source: Glob Health Sci Pract. 2022 Feb 28;10(1):e2100534. doi: 10.9745/GHSP-D-21-00534 (PMC8885352; doi:10.9745/GHSP-D-21-00534)
Supplement: GHSP-D-21-00534-Supplement.pdf [file GHSP-D-21-00534-Supplement.pdf]

Supplement to: Nai D, Tobey E, Fuseini K, Kuma-Aboagye P, Jain A. What factors influence self-injection of subcutaneous depot medroxyprogesterone acetate (DMPA-SC)? Results of a prospective cohort study in Ghana. *Glob Health Sci Pract*. 2022;10(1):e2100534. https://doi.org/10.9745/GHSP-D-21-00534

Supplement

Figure A1. Sankey diagram of patterns of mode of injection administration, discontinuation, and lost to follow up over the 3 injection periods (full sample, n=568 for each round)

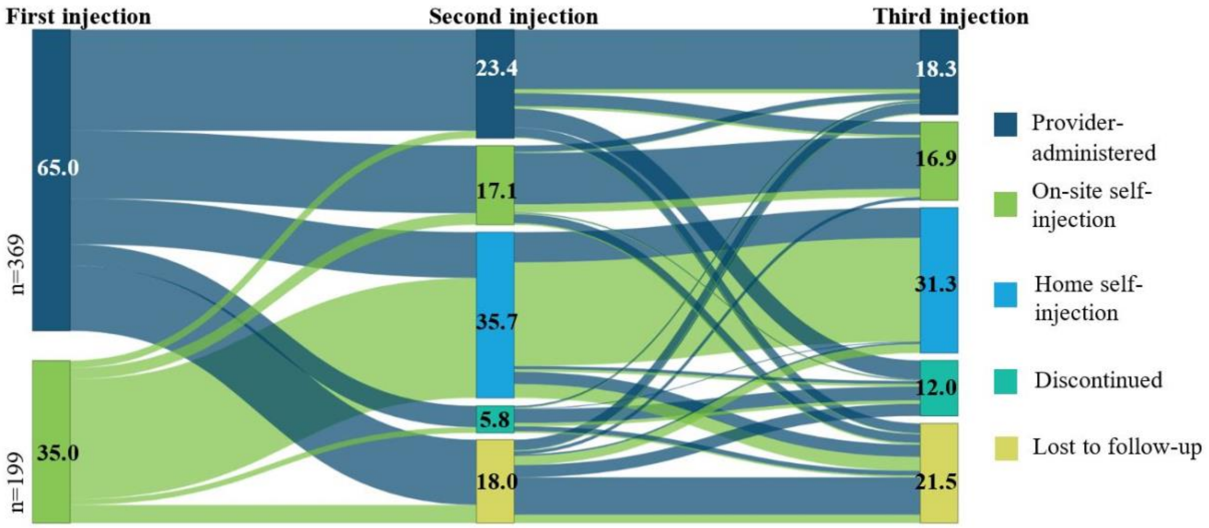

Supplement to: Nai D, Tobey E, Fuseini K, Kuma-Aboagye P, Jain A. What factors influence self-injection of subcutaneous depot medroxyprogesterone acetate (DMPA-SC)? Results of a prospective cohort study in Ghana. *Glob Health Sci Pract*. 2022;10(1):e2100534. https://doi.org/10.9745/GHSP-D-21-00534

Table. Adjusted odds ratios of being lost to follow-up at 6 months post DMPA-SC initiation compared to analytic study sample (n=499)^†

	Adjusted Odds Ratio	95% Confidence Interval
**Age range, years**		
18-24	ref	
25-29	1.13	(0.64, 1.97)
30-34	0.77	(0.37, 1.58)
35 and above	0.58	(0.28, 1.23)
**Marital status**		
Ever married/in union	ref	
Never married	0.43*	(0.23, 0.82)
**Education**		
No education	ref	
Primary	0.97	(0.41, 2.29)
JSS/JHS	0.79	(0.37, 1.71)
SSS/SHS or higher	0.56	(0.23, 1.35)
**Number of living children**		
No children	ref	
1-2 children	0.88	(0.40, 1.97)
3+ children	0.85	(0.34, 2.13)
**Employment status**		
Not currently working	ref	
Currently working	0.78	(0.48, 1.26)

* *P* <0.05.

^1 participant who was lost to follow-up was excluded from this analysis due to missing age.
†Adjusted for clustering at the region/residence level.
